# What makes a bad egg? Egg transcriptome reveals dysregulation of translational machinery and novel fertility genes important for fertilization

**DOI:** 10.1186/s12864-019-5930-8

**Published:** 2019-07-15

**Authors:** Caroline T. Cheung, Thao-vi Nguyen, Aurélie Le Cam, Amélie Patinote, Laurent Journot, Christelle Reynes, Julien Bobe

**Affiliations:** 1 INRA, Laboratoire de Physiologie et Génomique des poissons, Campus de Beaulieu, F-35042 Rennes cedex, France; 2 Institut de Génomique Fonctionnelle, IGF, Université de Montpellier, CNRS, INSERM, Montpellier, France; 3 Montpellier GenomiX, BioCampus Montpellier, MGX, Université de Montpellier, CNRS, INSERM, Montpellier, France

**Keywords:** Egg quality, Transcriptome, Microarray, Zebrafish, Differentially expressed genes, Prediction model

## Abstract

**Background:**

Egg quality can be defined as the egg ability to be fertilized and subsequently develop into a normal embryo. Previous research has shed light on factors that can influence egg quality. Large gaps however remain including a comprehensive view of what makes a bad egg. Initial development of the embryo relies on maternally-inherited molecules, such as transcripts, deposited in the egg during its formation. Bad egg quality is therefore susceptible to be associated with alteration or dysregulation of maternally-inherited transcripts. We performed transcriptome analysis on a large number (*N* = 136) of zebrafish egg clutches, each clutch being split to monitor developmental success and perform transcriptome analysis in parallel. We aimed at drawing a molecular portrait of the egg in order to characterize the relation between egg transcriptome and developmental success and to subsequently identify new candidate genes involved in fertility.

**Results:**

We identified 66 transcript that were differentially abundant in eggs of contrasted phenotype (low or high developmental success). Statistical modeling using partial least squares regression and genetics algorithm demonstrated that gene signatures from transcriptomic data can be used to predict developmental success. The identity and function of differentially expressed genes indicate a major dysregulation of genes of the translational machinery in poor quality eggs. Two genes, o*tulina* and *slc29a1a,* predominantly expressed in the ovary and dysregulated in poor quality eggs were further investigated using CRISPR/Cas9 mediated genome editing. Mutants of each gene revealed remarkable subfertility whereby the majority of their eggs were unfertilizable. The Wnt pathway appeared to be dysregulated in the *otulina* mutant-derived eggs.

**Conclusions:**

Here we show that egg transcriptome contains molecular signatures, which can be used to predict developmental success. Our results also indicate that poor egg quality in zebrafish is associated with a dysregulation of (i) the translational machinery genes and (ii) novel fertility genes, *otulina* and *slc29a1a,* playing an important role for fertilization. Together, our observations highlight the diversity of the possible causes of egg quality defects and reveal mechanisms of maternal origin behind the lack of fertilization and early embryonic failures that can occur under normal reproduction conditions.

**Electronic supplementary material:**

The online version of this article (10.1186/s12864-019-5930-8) contains supplementary material, which is available to authorized users.

## Background

Good quality or developmentally competent fish eggs are defined as those that are successfully fertilized and develop normally as viable, non-malformed embryos [1]. However, the detailed mechanisms that are involved in egg quality are still poorly understood, and at present, no predictive markers of egg quality exist. Maternal genes are those that produce factors that are involved in oocyte growth and in the earliest stages of embryonic development, including fertilization, parental genome union, and cell division. Since initial development of the embryo relies on these maternally-inherited molecules including coding and non-coding mRNAs and proteins that are deposited into the developing oocyte, thus, they would likely reflect egg quality [2, 3]. Among these, the maternally-provided transcriptome of the egg is critical in supporting early embryogenesis because transcription from the zygotic genome does not start until the mid-blastula transition (MBT) which occurs approximately 3–4 h post-fertilization (hpf) in zebrafish [4, 5] even though transcription starts earlier for “first wave” zygotic genes [6].

Previous research using both traditional mutational assays [7] as well as more recent transcriptomic analyses have revealed several maternal factors that can influence egg quality. The nucleoplasmin 2 (*npm2a* and *npm2b*) genes were recently found to be crucial for egg quality; suppression of *npm2b* resulted in embryonic arrest before zygotic genome activation (ZGA) in mouse and zebrafish, and *npm2a* deficiency in zebrafish led to a complete lack of embryonic development [8, 9]. Further, post-ovulatory ageing induced egg quality defects are associated with low mRNA levels of *igf1* (insulin growth factor 1) and beta-tubulin, as well as a small but significant overabundance of keratins 8 and 18, cathepsin Z, and *pgs2* (prostaglandin synthase 2) [10, 11]. In addition, controlled induction of ovulation by hormonal or photoperiod manipulation negatively impacts egg quality in rainbow trout, and the abundance of several genes including *apoC1* (apoliprotein C1), *mr-1* (major histocompatibility class 1 related protein), *ntan1* (N-terminal asparagine amidase 1), *myo1b* (myosin 1b), *pyc* (pyruvate carboxylase), as well as *phb2* (prohibitin 2) was found to be significantly different between eggs that were naturally and artificially spawned [12]. Other studies have suggested that genes involved in immune regulation have an impact on egg quality whereby variable abundance of transcripts in the interferon pathway and *mhc* (major histocompatibility) class genes was demonstrated in eggs of different quality [13, 14]. However, despite these results, knowledge on the factors that contribute to the quality of fish eggs remains patchy. Thus, in this study, we carried out a large-scale analysis to compare the transcriptome of one-cell stage eggs of different quality and performed statistical modeling of differentially expressed genes (DEGs) with survival in order to determine if there are common factors that impact egg quality in wildtype (WT) females that can then serve as markers and/or predictors of developmental competence. Our findings provide evidence that in different quality eggs from wildtype couples bred under standard conditions, gene signatures exist in the egg transcriptome, which can be used to predict developmental success. The identity and function of differentially expressed genes indicate a significant dysregulation of genes of the translational machinery. We further conducted functional analyses on two candidate genes that were dysregulated in bad quality eggs using the CRISPR/Cas9 knockout system and reveal for the first time the essential roles of two new potential fertility-related genes, *otulina* (OTU deubiquitinase with linear linkage specificity a) and *slc29a1a* (solute carrier family 29, member 1a) that appear to be important for fertilization. This dramatic decrease in the ability of the egg to be fertilized was associated with the dysregulation of the Wnt pathway in the case of *otulina*. Together, our observations indicate that poor zebrafish egg quality is associated with a dysregulation of (i) the translational machinery genes and (ii) novel fertility genes, *otulina* and *slc29a1a,* playing an important role for fertilization.

## Results

### Transcriptomic differences between good and bad eggs in all samples

**Fig. 1:**
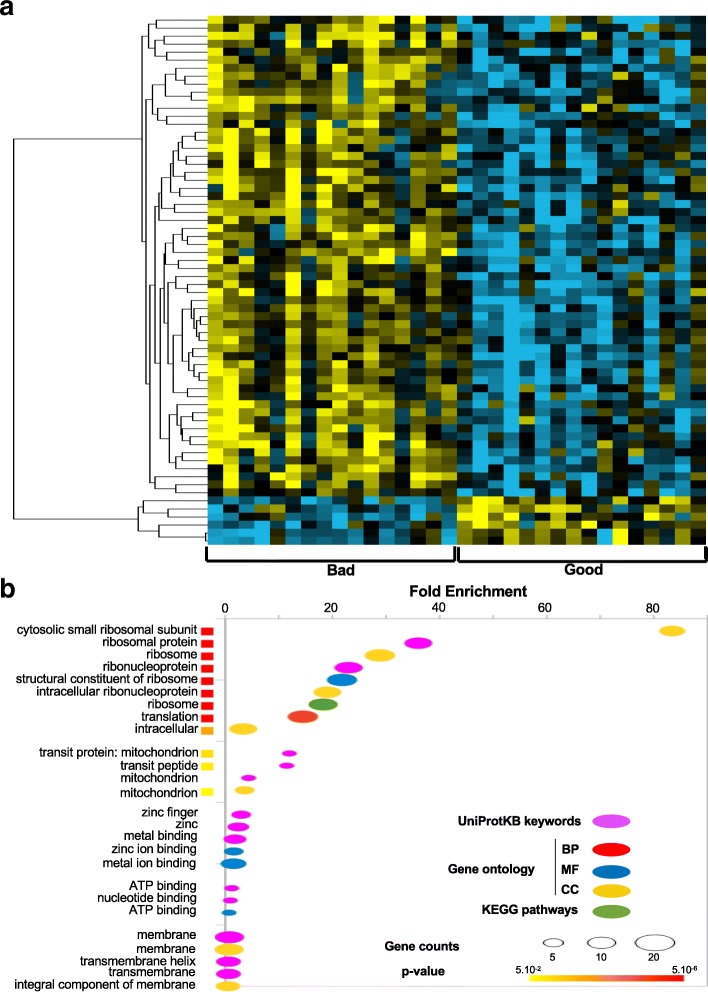
**a**: Heat map showing unsupervised clustering of the 66 differentially expressed genes (DEGs) between good and bad quality eggs from 32 clutches of fertilized zebrafish eggs. Yellow signal denotes upregulation, blue signal denotes downregulation, and black defines no change in expression. **b**: Gene ontology analysis using the DAVID online program of the 55 DEGs with known information. The enriched terms are shown on the y-axis and the fold enrichment is on the x-axis. Annotated terms are derived from UniProtKB keywords (pink circle); Gene Ontology BP (red circle), MF (blue circle), and CC (yellow circle); and KEGG pathways (green circle). Statistical significance is represented by the colored squares next to the enriched terms

Among the 136 egg clutches that we collected, we selected 16 clutches each of good and bad quality eggs defined as those with > 93 and < 38% survival at 48 hpf, respectively, for microarray analysis using a customized chip containing 61,657 annotated sequences of the zebrafish transcriptome. Only sequences for which a signal was measured in at least 80% of the samples from one experimental group were kept for further analysis, which was thus conducted on 31,317 annotated sequences. A T-Test (Benjamini-Hochberg (BH) corrected pval < 0.05) was used to determine which genes showed a significantly different level of expression between good and bad quality eggs. This statistical analysis led to the identification of 66 differentially expressed genes (DEGs, Additional file 1). We observed in the heat map showing unsupervised clustering (Fig. 1a) of the 66 DEGs that a majority of them were upregulated (60 genes, yellow signal) with only 6 genes that were down-regulated (blue signal) in bad quality eggs as compared to good quality eggs. Additional file 1 lists the 66 DEGs including their associated information. Of these 66 genes, 8 were referenced in Ensembl but not annotated (i.e. not associated with any known gene or protein).

### Overrepresentation analyses of gene ontological terms of the DEGs

We submitted the 66 DEGs to Gene Ontology functional annotation analysis using the online program, DAVID [15] with the zebrafish genes in the microarray set as background (Fig. 1b). Among the 66 DEGs submitted, functional terms associated with 55 annotated genes were identified and therefore classified by the DAVID program using terms derived from the following resources: UniProtKB keywords (pink circle); Gene Ontology Biological Process (red circle), Molecular Function (blue circle), and Cellular Component (yellow circle); and KEGG pathways (green circle). The analysis revealed 2 significant annotation clusters; in the first cluster (enrichment score: 9.98), the terms related to ‘ribosome’, ‘translation’, and ‘intracellular’ were enriched between 3- to 83-fold, while the second cluster included terms associated with ‘mitochondrial transit protein’, which were enriched between 3- to 12-fold. The genes that most drastically changed expression (i.e. ribosome production factor 2 homolog (*S. cerevisiae*) [*rpf2*], ribosomal protein S27 (isoform 2) [*rps27.2*], and U1 spliceosomal RNA [*U1*] with fold changes of 7.81, 1.90, and − 2.33/− 2.35, respectively) are associated with ‘translation’ and ‘ribosomes’.

### Quantitative real-time polymerase chain reaction (qPCR) validation of the DEGs

**Fig. 2:**
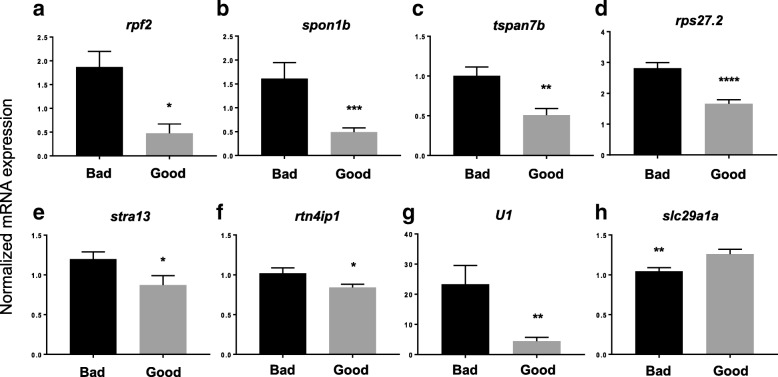
Validation of the microarray data by performance of quantitative real-time PCR (qPCR). Eight genes, including (**a**) *rpf2*, (**b**) *spon1b*, (**c**) *tspan7b*, (d) *rps27.2*, (**e**) *stra13*, (**f**) *rtn4ip1*, (**g**) *U1*, and (**h**) *slc29a1a* were subjected to qPCR using the primers listed in Additional file 2, whereby LSM couples member 14B (*lsm14b*), prefoldin subunit 2 (*pfdn2*), and ring finger protein 8 (*rnf8*) as well as 18S rRNA, *beta-actin* (*bact*), and *elongation factor 1 alpha* (*EF1α*) were used as internal controls. * *p*-value ≤0.05, ** *p*-value ≤0.01, *** *p*-value ≤0.001, **** *p*-value<< 0.001

In order to confirm the results obtained by microarray analysis, another independent method to detect gene expression changes was performed. qPCR was conducted using the same 32 samples that were submitted to microarray analysis and the primers used are listed in Additional file 2. Eight genes that underwent the most drastic changes in microarray analysis were subjected to qPCR, and their biological function as well as the *p*-value and fold change in the microarray analysis are shown in Additional file 3. qPCR confirmed that the expression of *rpf2* (1.87 ± 0.33 vs. 0.48 ± 0.20, *p* = 0.01), *spon1b* [spondin 1b] (1.61 ± 0.34 vs. 0.49 ± 0.09, *p* = 0.0003), *tspan7b* [tetraspanin 7b] (1.00 ± 0.11 vs. 0.50 ± 0.08, *p* = 0.001), *rps27.2* (2.82 ± 0.18 vs. 1.66 ± 0.13, *p* < 0.0001), *stra13* [stimulated by retinoic acid 13 homolog/centromere protein X] (1.20 ± 0.09 vs. 0.87 ± 0.12, *p* = 0.03), and *rtn4ip1* [reticulon 4 interacting protein 1] (1.02 ± 0.07 vs. 0.84 ± 0.04, p = 0.03) was increased in bad quality eggs as compared to good quality eggs, while that of *U1* (21.08 ± 5.81 vs. 4.38 ± 1.28, *p* = 0.009) and *slc29a1a* (1.04 ± 0.05 vs. 1.26 ± 0.06, *p* = 0.008) were increased in bad relative to good quality eggs (Fig. 2a-h). Interestingly, despite the statistical significance in the differential regulation of *U1* (Fig. 2g), the expression of this gene was regulated in the opposite direction by qPCR as compared to by microarray analysis. In fact, we found that *U1* expression was decreased on average by 2.3-fold in bad quality eggs relative to good quality eggs as assessed by microarray, but qPCR results showed that it was increased by approximately 5-fold in bad as compared to good quality eggs. Regardless of this difference, we found by both microarray and qPCR that the transcript levels of all eight genes were differentially regulated.

### Functional analysis of otulina and slc29a1a in zebrafish

**Fig. 3:**
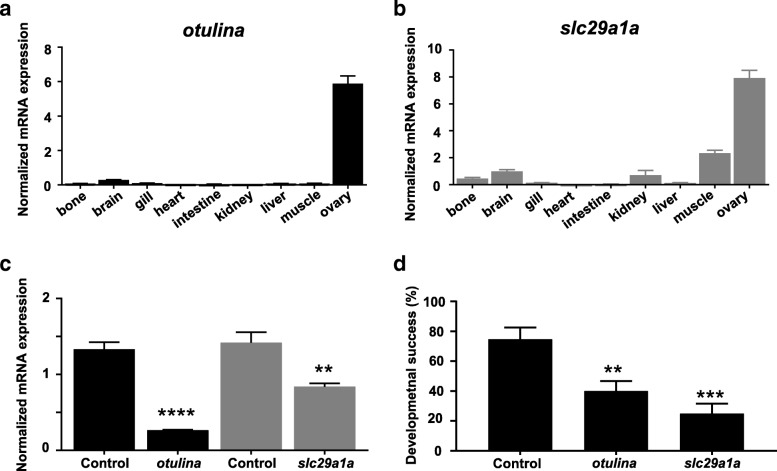
Tissue localization of *otulina* (**a**) and *slc29a1a* (**b**) based on qPCR assays. **c**: Expression level of *otulina* and *slc29a1a* in spawned eggs from mutant females mated with WT males as assessed by qPCR. 18S rRNA, *beta-actin* (*bact*), and *elongation factor 1 alpha* (*EF1α*) were used as internal controls, and experiments performed in triplicate. **d**: Developmental success in terms of survival rate of embryos at 24 h post-fertilization (hpf) from *otulina-* and *slc29a1a-*deficient mutant females mated with WT males. *N* = 4 r different females for *otulina* and *N* = 10 different females for *slc29a1a*, using eggs from at least three spawns for each individual female

In order to validate the in vivo significance of some of the DEGs, we performed functional analysis by genome editing using the CRISPR/Cas9 system. We first examined the tissue localization of our validated differentially abundant transcripts, *rpf2*, *spon1b*, *tspan7b*, *rps27.2*, *stra13*, *rtn4ip1*, and *slc29a1a* as well as *otulina*, which tended to be over abundant (*p* < 0.1) in good quality eggs, using RNA-seq data stored in the PhyloFish [16] online database (Additional file 4). We observed that only *slc29a1a* and *otulina* were predominantly expressed in ovary, egg, and/or embryo, thus, functional knockouts of these genes would mostly affect the female reproduction and the ensuing embryogenesis with minimal effect on non-reproductive organs. qPCR analysis for *otulina* (Fig. 3a) and *slc29a1a* (Fig. 3b) in different zebrafish tissues confirmed that both of these genes were expressed predominantly in the ovary and therefore good candidates for knockdown. One-cell stage embryos were injected with the CRISPR/Cas9 guides that targeted either *otulina* or *slc29a1a.* Injected embryos were subsequently raised until sexual maturity. Mosaic founder mutant females (F0) were identified by fin clip genotyping and subsequently mated with wild-type (WT) or Dr_*vasa*:eGFP C3 (hereafter called *vasa*:eGFP) males, and embryonic development was recorded. While mutations could be detected in F0 females, these mutations were not transmitted to the next generation thus making it impossible to generate non-mosaic homozygote mutant fish for further analysis. Since the mutagenesis efficiency of the CRISPR/Cas9 system was very high [17, 18], the *otulina* and *slc29a1a* genes were however sufficiently knocked-out in the transgenic mosaic F0 females to study the phenotype associated with the partial knock out of target genes, as previously shown for other maternal genes in zebrafish [9, 19]. This was evidenced by the substantially lower transcript levels of *otulina* and *slc29a1a* in the eggs of these mutant mosaic females as compared to those from control WT pairings (Fig. 3c). The decrease in maternally inherited transcript levels was especially high in *otulina* mutants. Thus, the phenotypes of *otulina* (*n* = 4) and *slc29a1a* (*n* = 10) mutants could be observed even in the F0 generation. All of our observations were therefore obtained from the F0 generation using eggs from mosaic mutant females in which target genes were partially knocked out and corresponding expression significantly reduced in eggs.

**Fig. 4:**
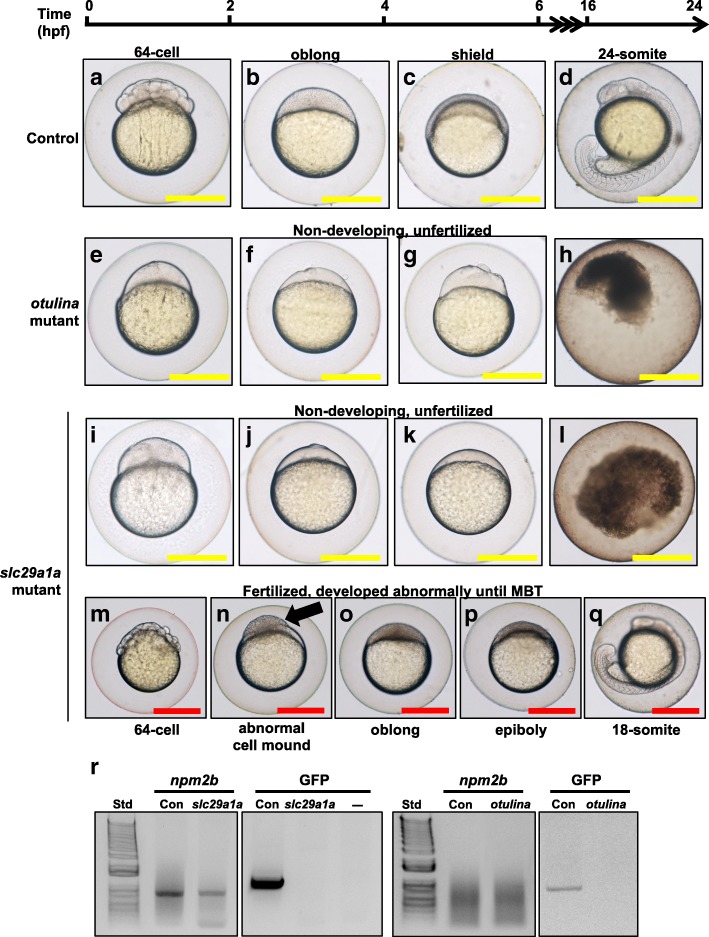
Representative images showing the development between 0 and 24 h post-fertilization (hpf) of F1 embryos from wildtype control (**a**-**d**), *otulina-*deficient (**e**-**h**), and *slc29a1a*-deficient (**i**-**q**) females. In the control eggs, the embryos were at 64-cell (A), oblong (**b**), shield (**c**), and 24-somite (**d**) stages according to Kimmel et al. [56]. Eggs from *otulina* and *slc29a1a* mutant females were non-developing and did not under any cell division (E-L). Some eggs from two *slc29a1a* mutant females were developing abnormally (M-Q). (**a**, **e**, **i**, **m**) = images taken at 2 hpf; (**b**, **f**, **j**, **n**) = images taken at 4 hpf; (**c**, **g**, **k**, **o**) = images taken at 6 hpf; (P) = image taken at 8 hpf; (**d**, **h**, **l**, **q**) = images taken at 24 hpf. The arrow demonstrates a partially cellularized blastodisc that was sitting atop an enlarged syncytium. Scale bars denote 500 μm. R: PCR genotyping for *nucleoplasmin 2b* (*npm2b*) and *vasa:eGFP* in spawned eggs from WT, *otulina-*, and *slc29a1a-*mutant females crossed with *vasa:eGFP* males to detect fertilization of the eggs. Std = 1 kb ladder; Con = WT female crossed with *vasa:eGFP* male

We observed that both *otulina* and *slc29a1a* mutant-derived eggs had a significantly lower developmental success, defined as the proportion of surviving embryos at 24 hpf to the total number of spawned eggs (40.0 ± 6.7% and 24.8 ± 6.8%, respectively) in comparison to controls (74.61 ± 7.9%) (Fig. 3d). Eggs from the cross between each individual mutant female and a *vasa:eGFP* male were counted based on their developmental phenotype, described as non-cellularized (lack of cell division), partially cellularized (abnormal cell division), and normal development, as shown in Table 1. As compared to the control embryos that developed normally from 2 to 24 hpf (Fig. 4a-d), most of the spawned eggs from the mutant females were non-cellularized such that they did not undergo any cell division at all throughout the same time period, and they eventually all died by 24 hpf (Fig. 4e-l). However, two of the *slc29a1a* mutants displayed some heterogeneity in their offspring; while a proportion of the spawn did not develop and did not undergo cell division as observed previously, a number of cells underwent abnormal development characterized by asymmetrical cell division and the appearance of a cell mound on top of an enlarged cytoplasm, which occurred until approximately 4–5 hpf (Fig. 4m and n), after which they began to develop normally albeit slightly slower than their control counterparts (Fig. 4o-q). To determine if the non-cellularized eggs were unfertilized or were arrested in development immediately after fertilization, we performed PCR genotyping for the *gfp* gene, which would only come from the *vasa:eGFP* male and not from the mutant mother that does not harbour any *gfp* gene. We found that the non-cellularized eggs from both *otulina* and *slc29a1a* mutants did not have the *gfp* gene indicating that they were not fertilized (Fig. 4r).

**Table 1:** Characterization of *otulina* and *slc29a1a* mutant phenotypes

Embryos with defects				
	Total number of embryos	Non-cellularized^†^	Partially cellularized ^‡^	Normal embryos
*otulina-1*	210	163		47
*otulina-2*	213	172		41
*otulina-3*	92	53		39
*otulina-4*	116	110		6
*slc29a1a-1*	104	104		0
*slc29a1a-2*	100	88		12
*slc29a1a-3*	660	450		210
*slc29a1a-4*	451	439		12
*slc29a1a-5*	245	150		95
*slc29a1a-6*	152	138		14
*slc29a1a-7*	361	252		110
*slc29a1a-8*	80	71		9
*slc29a1a-9*	85	9	48	28
*slc29a1a-10*	370	153	24	193

Characterization of *otulina* and *slc29a1a* mutant phenotypes from crosses of *otulina* or *slc29a1a* mutant F0 females and WT males. ^†^Embryos did not develop at all. ^‡^Embryos had a partially cellularized blastodisc that was sitting atop an enlarged syncytium (arrow in Fig. 4n)

### The Wnt pathway is dysregulated following otulina deficiency

**Fig. 5:**
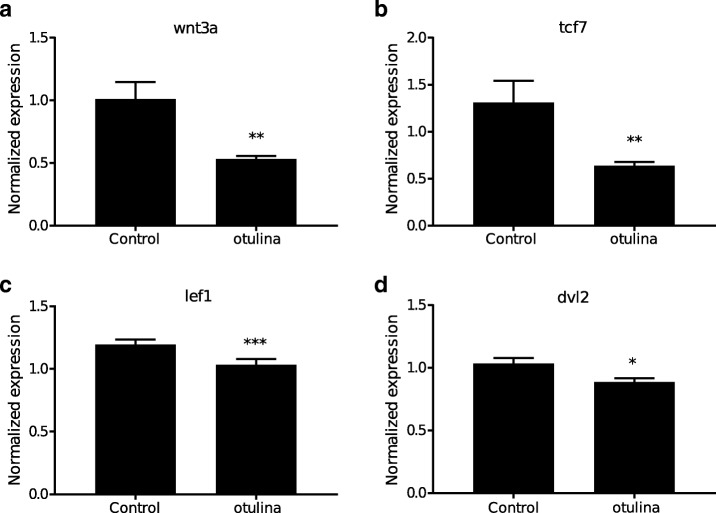
Evaluation of the expression levels (arbitrary units) of *wnt3a* (**a**), *tcf7* (**b**), *lef1* (**c**), and *dvl2* (**d**) in spawned eggs from *otulina-*deficient mutant females mated with WT males as assessed by qPCR. 18S rRNA, *beta-actin* (*bact*), and *elongation factor 1 alpha* (*EF1α*) were used as internal controls, and experiments performed in triplicate. N = 4 different females, at least three spawns from each female

In a bid to elucidate a possible mechanism that may govern the function of *otulina*, we assessed the spawned eggs from *otulina*-mutant females crossed with WT males for the transcript levels of components of the *wnt* (*wnt3a*, *tcf3*, *tcf7*, *lef1*, and *dvl2*) and *tnf*/*nf-κb* (*nf-kb2*, *rel*, *rela, ikkaa*, *ikkab*, and *tnfa*) pathways as *otulina* plays a role in these pathways in mammalian models [20–22]. Our findings showed that *wnt3a*, *tcf7*, *lef1*, and *dvl2*, but not *tcf3*, transcript levels were significantly decreased in the *otulina* mutant-derived eggs (Fig. 5a-d), while none of the transcripts belonging to the *tnf*/*nf-κb* pathways exhibited a change in transcript levels (Additional file 5).

### Identification of gene signatures to predict developmental competence by statistical modeling using partial Least Square (PLS) regression and genetic algorithm (GA)

**Fig. 6:**
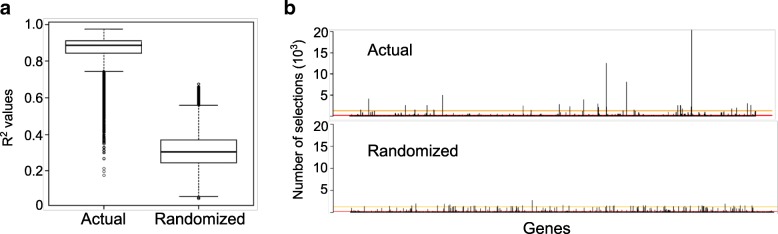
**a** The average 2-fold cross validation R^2^ values obtained from the actual dataset were compared to the ones obtained from the pseudo-datasets with permuted survival rates. **b**: The frequency that each variable was selected in populations from the actual data and from the randomized data. The 95th and 99th percentiles of the distribution of frequencies in the randomized data were used to obtain sets of genes that were the most often selected

Findings reported above in the transcriptome analysis were based on univariate analysis. We additionally used PLS to model the link between transcriptomic data and survival rates, and a GA to select subsets of genes that best predicted survival rates (see Methods for details). We ran the PLS-GA procedure with 70 populations of 500 potential solutions, i.e. subsets of 1 to 20 randomly selected genes. We thus obtained 35,000 final potential solutions, which we evaluated by 10 runs of 2-fold cross validation (2-FCV); we defined the average cross-validated R^2^ values from the 10 runs as the quality criterion for each individual. To confirm that the selected individuals were relevant, we compared the average 2-FCV R^2^ values obtained on the actual dataset to the ones obtained on data generated by randomly permuting the survival rates of the different observations. Figure 6a shows that the 2-FCV R^2^ obtained for the final individuals using the actual survival rates were significantly higher than those obtained with the randomized data (*p*-value < 2.10^− 16^; Mann-Whitney U-test). In addition, we compared the distribution of selection frequencies of each gene in the final populations using the actual and randomized data. We did not observe significant peaks (genes with high selection frequency) in the randomized data (Fig. 6b, lower panel) as compared to the actual data (Fig. 6b, upper panel). The 95th and 99th percentiles of the distribution of frequencies in the randomized data were then used as thresholds to identify sets of genes that were significantly frequently selected. We identified 156 genes using the 95th percentile. Table 2 displays the 29 genes identified using the 99th percentile. Of note, 10 of the 29 genes were identified as differentially expressed in the microarray analysis. A gene signature with a low number of genes is desirable for diagnostic or prediction purpose. Table 3 displays the two best solutions with only 7 and 8 genes and average 2-FCV R^2^ values equal to 0.9771 and 0.9678, respectively. In conclusion, we identified statistically robust gene signatures that predicted egg survival rates from transcriptomic data.

**Table 2:** Genes identified from the PLS-GA analysis to be associated with survival

ENSEMBL gene annotation	Gene name
ENSDARG00000090871	Si:dkey-210j14.4
ENSDARG00000076419	Si:dkeyp-117b11.2
ENSDARG00000079255	Zgc:174935
*ENSDARG00000031366*	***Reticulon 4 interacting protein 1***
ENSDARG00000006982	muscle segment homeobox D
ENSDARG00000071553	Zgc:171500
ENSDARG00000070898/ENSDARG00000092291	Si:ch211-262 h13.3 / Si:ch211-281 g2.3
ENSDARG00000075318	Solute carrier family 16 (monocarboxylic acid transporters), member 6a
*ENSDARG00000063295*	***Myosin, heavy polypeptide 9a, non-muscle***
*ENSDARG00000082140/ENSDARG00000082017*	***U1 spliceosomal RNA***
ENSDARG00000089078	Collagen, type XXIII, alpha 1
*ENSDARG00000017820*	***Polymerase (RNA) III (DNA directed) polypeptide D***
ENSDARG00000024687	Polymerase (RNA) III (DNA directed) polypeptide G
*ENSDARG00000089422*	***CABZ01087562.1***
ENSDARG00000056563	Peroxisome proliferative activated receptor, gamma, coactivator 1, beta
*ENSDARG00000076498*	***Golgi integral membrane protein 4a***
*ENSDARG00000069425*	***Heat shock factor binding protein 1a***
ENSDARG00000090804	G protein-coupled receptor 155a
ENSDARG00000075434	RNA 2,3,-cyclic phosphate and 5,-OH ligase
ENSDARG00000096436	Si:dkey-118j18.4
ENSDARG00000089677	CABZ01117603.1
*ENSDARG00000095796*	***Si:dkey-87o1.2***
ENSDARG00000004898	Zona pellucida glycoprotein 2, like 2
ENSDARG00000020149	Acyl-Coenzyme A oxidase-like
ENSDARG00000027738	Si:ch211-13c6.2
ENSDARG00000080245	5S ribosomal RNA
ENSDARG00000058445	Protein disulfide isomerase-like, testis expresse
*ENSDARG00000078785*	***Transmembrane protein 258***
*ENSDARG00000093926/ENSDARG00000095522*	***Si:dkey-71b5.2 / Si:dkey-71b5.3***

List of the 29 genes that were selected from the exhaustive analysis by Partial Least Square (PLS) regression and genetic algorithm (GA). The 10 genes that were differentially regulated in our microarray dataset are boldfaced and italicized

**Table 3:** Two solutions from the parsimonic prediction model

ENSEMBL gene ref.	Gene name
Solution 1	
*ENSDARG00000079255*	*Zgc:174935*
*ENSDARG00000089677*	*CABZ01117603.1*
*ENSDARG00000090871*	*Si:dkey-210j14.4*
*ENSDARG00000076419*	*Si:dkeyp-117b11.2*
*ENSDARG00000017820*	*polymerase (RNA) III (DNA directed) polypeptide D*
ENSDARG00000086485	novel protein coding gene
ENSDARG00000020054	aldehyde oxidase 1
Solution 2	
*ENSDARG00000079255*	*Zgc:174935*
*ENSDARG00000089677*	*CABZ01117603.1*
*ENSDARG00000090871*	*Si:dkey-210j14.4*
*ENSDARG00000076419*	*Si:dkeyp-117b11.2*
*ENSDARG00000017820*	*polymerase (RNA) III (DNA directed) polypeptide D*
ENSDARG00000016855	Splicing factor 3b, subunit 5
ENSDARG00000088305	CABZ01072929.1
ENSDARG00000087431	Zgc:173962

Two solutions containing 7 and 8 genes that were selected from the parsimonic model by Partial Least Square (PLS) regression and genetic algorithm (GA). The 5 common genes between the two solutions are italicized

## Discussion

In this study, we identified 66 DEGs between good and bad quality eggs at the one-cell stage, which is a relatively low number as compared to other studies. In fact, it must be reiterated that all of the couples that were mated and produced clutches were wildtype without any particular treatment in contrast to most existing studies on fish egg quality. Thus, there may have been multiple natural causes behind the decline in quality of the eggs from the different mothers, such as nutrition, density, age of parents, delay from last spawn, and genetics just to name a few [1, 23, 24]. Among the 66 DEGs, 7 genes were verified independently by qPCR. These genes all play different cellular roles: *rpf2* [25] is a ribosome assembly protein that recruit 5S rRNA and ribosomal proteins into nascent large ribosome subunits; *rps27.2* [26] is a structural component of the 40S small ribosome subunit; *spond1b* [27] encodes a protein secreted by floor plate cells during embryogenesis that localizes to the central spinal canal and has neuroregulatory functions; *rtn4ip1* [28] is a mitochondrial protein present in neurons and astrocytes; *tspan7b* [29] is a cell surface receptor signaling molecule that functions in embryonic development; *stra13* [30] has roles in DNA repair and kinetochore assembly; and *slc29a1a* [31] is transmembrane glycoprotein that mediates the cellular uptake of nucleosides. Their distinct roles in the cell highlight the fact that embryonic survival is based on many different cellular processes and suggest that they may serve as candidate markers of egg quality among unrelated wildtype females in larger populations.

With regard to the functional characteristics of the DEGs in the study using all samples, overrepresentation analyses of the GO terms by the DAVID gene ontology program found that genes that function in ribosome and translation were predominantly enriched. A recent study demonstrated that ribosome was one of the top over-represented KEGG pathways in the expressed transcriptome of unfertilized zebrafish eggs, which suggests that the ribosome/translation process plays a major role in fertilization and subsequent embryonic development [32]. Further, our results are consistent with previous findings that showed that translation-related transcripts were also differentially expressed in seabass eggs of different quality [33]. Interestingly, the findings in this study correlate with our previous proteomic study which also demonstrated significant dysregulation of proteins with functions in protein synthesis in zebrafish eggs of varying quality [34]. In the proteomic study, peptides that function in protein synthesis were upregulated in both good and bad quality eggs, which suggests a general dysregulation of the system. In this study, *rpf2* and *rps27.2* were found by microarray and confirmed by qPCR to be increased in bad quality eggs (Fig. 2a and d, respectively). Both of these genes encode proteins that function in ribosomes; rpf2 is an assembly factor and rps27.2 is a structural component of the 40S small ribosome subunit as mentioned above. A similar finding was demonstrated in a previous study that investigated the transcriptome of eggs after natural and controlled ovulation in rainbow trout (*Oncorhynchus mykiss*); it was revealed that *rpl24 transcript*, which encodes a structural component of the large ribosome subunit, was more abundant in the latter which had higher mortality [12]. Thus, it appears that eggs of bad quality are associated with a dysregulation of genes encoding ribosome components. Dysregulation of the translational machinery in zebrafish eggs of different quality appears to be at both the transcript and protein levels, and may disrupt developmental competence and impact egg quality. Whether or not this is simply a consequence of the dysregulation of the egg formation process in the ovary or the reason why bad quality eggs have a lower ability to develop once fertilized is currently unknown and begs for further investigations.

Obtaining a gene signature to predict the survival rate is valuable and of practical interest as the identification of a set of genes that correlates with the rate of survival can open up avenues for understanding the biological phenomena to explain egg quality and for future applications in aquaculture [35]. Existing studies, that used machine learning to predict egg quality resulted in the identification of several hundreds of genes [36]. In the present study, we used PLS to model the link between transcriptomic data and survival rates, and a genetic algorithm to select subsets of genes that best predicted survival rates. To our knowledge, such an approach had never been applied to egg quality in fish. We succeeded in identifying statistically robust gene signatures with low number of genes (< 10) that predicted egg survival rates from transcriptomic data.

In an effort to investigate the functional significance of some of the DEGs, we created CRISPR/Cas9 knockouts of *otulina* and *sc29a1a* due to their ovarian-predominant expression and dysregulation in bad quality eggs (Additional file 4, Fig. 2). We used the F0 mosaic females directly for experimentation since the *otulina* and *slc29a1a* transcript levels were dramatically reduced in their F1 eggs, which indicated that many of the oocytes in the mosaic females contained the mutant copy of the gene and thus had reduced gene expression. Our findings provide evidence that *otulina* and *slc29a1a* are key fertility-related genes playing an important role for the production of fertilizable eggs. Notably, we demonstrated for the first time that deficiency in each of these genes render females subfertile, with complete lack of development in the spawned eggs, which were shown to be unfertilized (Fig. 4r). Thus, our data suggested that *otulina* and *slc29a1a* may play roles that contribute to the factors important for fertilization. *Otulina* is predicted to encode for a deubiquitinase, which removes methionine 1-linked ubiquitin chains, of the OTU family in zebrafish, and substrate-bound otulin in mammals has been shown to associate with the linear ubiquitin chain assembly complex (LUBAC). This ubiquitination-deubiquitination system is a key regulator of important signaling pathways, including Wnt, TNF-α, and NF-κb. The *otulin* gene, the mammalian ortholog, has been previously shown to be involved in early development in mice since a functionally-disruptive gene mutation results in embryonic lethality due to perturbed Wnt signaling and angiogenesis [20]. In fact, it is known that Wnt signaling plays a major role in gonad differentiation in some fish species [37–39] . Further, *otulin* has also been shown to be a key factor in regulating inflammation and immunity through its modulatory role in the TNF-α and NF-κb pathways [21, 22]. It is known that inflammatory signaling is an essential part of early embryonic development since many of these components are part of the maternally-inherited repertoire of transcripts, and the TNF- α and NF-κb pathways play important roles in embryonic hematopoietic stem and progenitor cell production as well as body patterning/specification [40–42]. Our results showed that there were significant decreases in the transcript levels of several *wnt* components including *wnt3a*, *tcf7*, *lef1*, and *dvl2* (Fig. 5), while none of the transcripts belonging to the *tnf*/*nf-κb* pathways showed any changes. Thus, *otulina* deficiency may contribute to subfertility in zebrafish via dysregulation of *wnt* signaling, in line with our previous study that showed that the *wnt* pathway was disturbed at the protein level in bad quality eggs and with the known function of *wnt* in development [34, 43, 44].

On the other hand, *slc29a1a* is predicted to encode for an equilibrative nucleoside transporter. In mammals, it was shown that *slc29a1* transports adenosine, which is a potent cellular metabolite that functions in cyclic AMP pathways and also acts directly as a vasoactive mediator, into fetal cells and has implications in fetal endothelial functions such that its dysfunction can lead to human pregnancy-related problems such as gestational diabetes, intrauterine growth restriction, and pre-eclampsia [45–47]. In addition, *slc29* homologues in chicken play important roles in rhythm and conduction in developing embryonic hearts via the ERK/MAP (extracellular signal regulated kinase/mitogen activated protein) pathways [48]. However, the function of *slc29a1* in fish is still unknown since these species usually undergo external fertilization and embryonic growth. Further investigations into their physiological functions are warranted.

## Conclusions

In this report, we show that statistically robust gene signatures exist in the maternally-inherited transcriptome that could be used to predict development competence. We also report that poor zebrafish egg quality is associated with a dysregulation of the translational machinery genes. Finally, we identified novel fertility-related genes, *otulina* and *slc29a1a,* that play an important role to allow the production of fertilizable eggs.

## Methods

### Fish husbandry and sample collection

Wildtype zebrafish (*Danio rerio*) of the AB strain were maintained at 25 °C in a central filtration recirculating system with a 12 h light/dark cycle in the INRA LPGP fish facility (Rennes, France). Breeding pairs were kept in the same tank overnight separated by a partition, and in the morning, the divider was removed after which the female released her eggs to be fertilized by the male. One hundred and thirty-six clutches of fertilized zebrafish eggs at the one-cell stage were harvested and divided into two parts. One part was flash-frozen in TRI reagent (Sigma-Aldrich, St. Louis, USA) and stored at -80 °C for molecular biology analyses. The other part was cultured in modified Yamamoto’s embryo solution (17 mM NaCl, 400 μM KCl, 270 μM CaCl_2_.2H_2_O, 650 μM MgSO_4_.7H_2_O, 0.1 μl/ml of methylene blue) and monitored for up to 48 h, and the number of survivors was counted at 8, 24, and 48 hpf. Good quality eggs were defined as embryos that had a very high survival rate (> 93%) at 48 hpf and bad quality eggs were those that suffered a very low survival rate (< 38%) at 48 hpf. Those percentages were selected to obtain two groups of contrasted phenotype (i.e. differential egg quality) with sufficient statistical power.

### RNA extraction

Total RNA of the pooled clutches was extracted using TRI reagent according to the manufacturer’s protocol, and RNA quality and purity were assessed using the Agilent Nano RNA 6000 assay kit and 2100 Bioanalyzer (Agilent Technologies, Santa Clara, USA). All samples were confirmed to have a RIN (RNA integrity number) of 9–10 which are generally accepted as reflecting very good quality RNA.

### Microarray analysis

Zebrafish gene expression profiling was conducted using an Agilent 8x60K high-density oligonucleotide microarray. Labeling and hybridization steps were performed following the Agilent “One-Color Microarray-Based Gene Expression Analysis (Low Input Quick Amp labeling)” protocol. Briefly, for each sample, 150 ng of total RNA was amplified and labeled using Cy3-CTP. Yield (> 825 ng cRNA) and specific activity (> 6 pmol of Cy3 per μg of cRNA) of Cy3-cRNA produced were checked with the NanoDrop 2000 spectrophotometer (Thermo Fisher Scientific, Waltham, USA). 600 ng of Cy3-cRNA was fragmented and hybridized on a sub-array. Hybridization was carried out for 17 h at 65 °C in a rotating hybridization oven prior to washing and scanning with an Agilent Scanner (Agilent DNA Microarray Scanner, Agilent Technologies) using the standard parameters for a gene expression 8x60K oligoarray (3 μm and 20 bits). Data were then obtained with the Agilent Feature Extraction software (10.7.3.1) according to the appropriate GE protocol (GE1_107_Sep09) and imported into GeneSpring GX software (Agilent Technologies) for analysis. The data were first normalized by median centering, log-transformed, and and filtered considering expressed genes those which have a signal above background in at least 80% of the samples in at least one of the two conditions. Then differential expressed genes (DEGs) were determined by performing a T-Test with a Benjamini-Hochberg (BH) corrected pval < 0.05. Genes and biological samples were finally classified according to their gene expression profiles using a hierarchical clustering method (Cluster software) and the results were visualized with Treeview [49].

### Gene ontology (GO) analysis

The differentially expressed genes (DEGs) obtained from the microarray analysis were subjected to overrepresentation analyses using the DAVID version 6.7 (https://david.ncifcrf.gov/) [15] online program with Ensembl gene identifiers to elucidate enriched terms. The DAVID analyses were conducted using the Functional Annotation Tool based on terms derived from UniProtKB keywords, Gene Ontology BP, MF, and CC, and KEGG pathways with Benjamini multiple test correction (*p* < 0.05).

### Reverse transcription polymerase chain reaction and quantitative real-time PCR (qPCR)

One μg of RNA was used as template for synthesis of cDNA using the Maxima First Strand cDNA Synthesis Kit (Thermo Fisher Scientific) as per the manufacturer’s protocol. The cDNA samples were then diluted 20-fold and subjected to qPCR using the primers listed in Additional file 2. Primers were designed using the online program Primer3 (http://primer3.ut.ee) and extended across an intron when possible to eliminate the contribution from genomic DNA. qPCR was performed in triplicate using the GoTaq qPCR Mastermix kit (Promega, Madison, USA), which utilizes carboxy-X-rhodamine (CXR) as the reference fluorochrome, using the following cycling condition: 95 °C for 10 s and 60 °C for 30 s for 40 cycles. The data were collected with the StepOnePlus apparatus (Applied Biosystems, Foster City, USA) and quantitation of the samples was conducted using standard curves. LSM couples member 14B (*lsm14b*), prefoldin subunit 2 (*pfdn2*), and ring finger protein 8 (*rnf8*) had the most stable expression in the microarray dataset and were thus used as internal controls for qPCR. Further, 18S rRNA, *beta-actin* (*bact*), and *elongation factor 1 alpha* (*EF1α*) were also used as internal controls for qPCR [50]. The geometric means of all 6 genes were calculated and for normalization of the data quantity.

### CRISPR-Cas9 genetic knockout

CRISPR/Cas9 guide RNA (gRNA) were designed using the ZiFiT Targeter online software (version 4.2) [51, 52] and were made against 3 targets within each gene to generate large genomic deletions, ranging from 130 to 1500 base pairs, that span exons to induce the formation of non-functional proteins. The gRNA sequences for *otulina* are: 5′ GGAGACGCATGAGGATGAAC 3′; 5′ GGAAACAAACAGCATATTCT 3′; and 5′ GGGTCAGGTATCAAATAACT 3′. The gRNA sequences for *slc29a1a* are: 5′ GGGAGCCGCGTTATCCTT3’; 5′ GGGGCTTGTCAGAAACTA 3′; and 5′ TAGGAACCATATACAAAAAA 3′. Nucleotide sequences containing the gRNA were ordered, annealed together, and cloned into the DR274 plasmid. In vitro transcription of the gRNA from the T7 initiation site was performed using the Maxiscript T7 kit (Applied Biosystems), and their purity and integrity were assessed using the Agilent RNA 6000 Nano Assay kit and 2100 Bioanalyzer (Agilent Technologies). Zebrafish embryos at the one-cell stage were micro-injected with approximately 30–40 pg of each CRISPR/Cas9 guide along with 8–9 nM of purified Cas9 protein (a generous gift from Dr. Anne de Cian from the National Museum of Natural History in Paris, France). The embryos were allowed to grow to adulthood, and genotyped using fin clip and PCR that detected the deleted regions. The PCR bands of the mutants were then sent for sequencing to verify the deletion. Once confirmed, the mutant females were mated with wildtype males to produce F1 embryos, whose phenotypes were subsequently recorded. Images shown on Fig. 4 were captured with a AZ100 microscope and DS-Ri1 camera (Nikon, Tokyo, Japan).

### Genotyping by PCR

Genotyping of F0 and F1 animals was performed by harvesting fin clips from adult mosaic females as well as F1 animals under anesthesia (0.1% phenoxyethanol) and F1 eggs (1–2 cell stage) from mosaic females crossed with *vasa:eGFP* males. These samples were lysed with 5% chelex containing 100 μg of proteinase K at 55 °C for 2 h and then 99 °C for 10 min. The extracted DNA was subjected to PCR using the AccuPrime system (Promega) for *slc29a1a*, Advantage 2 system for *nucleoplasmin 2b* (*npm2b*), and Jumpstart Taq polymerase (Sigma-Aldrich) for *otulina* and *vasa:eGFP*. The primers are listed in Additional file 2.

### Statistical analyses

Statistical analysis of the difference in the expression of each gene between bad and good quality embryos was performed using a Student’s t-test whenever possible after determination of normality of distribution using the Anderson-Darling test. When a t-test could not be used, a Mann-Whitney’s U-test was used. All statistical determinations were conducted using Prism version 7 (GraphPad, La Jolla, USA). Data are presented as mean ± standard error (SEM). A *p*-value < 0.05 was considered as statistically significant.

### Analyses by partial least squares (PLS) regression and genetic algorithm (GA)

We first filtered uninformative genes by performing a correlation test between each gene expression levels and survival rate; genes with *p*-values > 0.1 were discarded. We then computed the Pearson correlation coefficient for the expression levels of each pair of genes. We considered genes with pairwise correlations higher than 0.95 as redundant and kept only the gene with the highest correlation to the survival rates in each subset of correlated genes.

We implemented a GA to select optimal individuals, i.e. subsets of genes whose expression levels predict survival rate. We generated the initial population of potential solutions by selecting 500 subsets of *p* genes (1 ≤ *p* ≤ 20) randomly among the 5410 filtered genes [53]. We applied PLS regression to each subset, which combined linearly the expression levels of the different genes of one subset to estimate the survival rate. We evaluated the quality of the different individuals using the squared Pearson correlation coefficient between the actual survival rates and the estimates [54]. We performed two-fold cross-validation (2-FCV) to prevent over-fitting; we randomly split the observations into two equal subsets d0 and d1. We applied the PLS model to d0 and assessed the quality of the model on d1, and vice versa. We calculated the squared Pearson correlation coefficient, 2-FCV R^2^, between the actual survival rates and the estimates obtained by the 2-FCV procedure. We ranked the 500 subsets according to the 2-FCV R^2^ values. We selected the best individuals by associating each individual with a selection probability that was proportional to its rank [55]. We altered the selected individuals through ‘mutation’ and ‘cross-over’. We mutated 90% of the individuals by randomly adding, removing or replacing a gene. We crossed-over 50% of the individuals by randomly splitting each of two potential solutions into two subsets of genes and exchanging one subset between them to obtain new individuals. We repeated this procedure for 200 generations and submitted the individuals selected in the final generation to extensive evaluation using 10 runs of 2-FCV. We used the average R^2^ obtained for each individual as the final criterion to quantify the quality of each final subset of genes.

To assess the relevance of the procedure, we applied the same GA to randomized datasets obtained by randomly permuting the survival rate of the different observations. Genes involved in egg survival are expected to appear often in the final subsets. The distribution of the selection frequency of each gene in the randomized datasets was computed. Genes with selection frequency in the actual data higher than the 95th or 99th percentile of the selection frequencies in the randomized data were considered as relevant. The distributions of the average 2-FCV R^2^ of the final individuals obtained on the actual and randomized data were also compared. The relationship between gene expression and survival rate was considered significant when the *actual* R^2^ values were significantly higher than the R^2^ values of the randomized dataset. To compare their distributions, a Mann-Whitney test was used.

## Additional Files

Additional file 1: The complete list of the 66 DEGs including the gene description, Ensembl annotation, corrected *p*-value, and fold change. Analysis was performed with the GeneSpring GX program. (XLSX 13 kb)

Additional file 2: Sequences of the primer pairs that were used in this study. (XLSX 13 kb)

Additional file 3: List of differentially expressed genes (DEGs) in bad quality eggs as compared to good quality eggs that were the most modified among the 66 DEGs found by microarray analysis. (XLSX 10 kb)

Additional file 4: Tissue localization of (a) *rpf2*, (b) *spond1b*, (c) *tspan7b*, (d) *rps27.2*, (e) *stra13*, (f) *rtn4ip1*, (g) *slc29a1a*, and (h) *otulina* transcripts by RNA-seq retrieved from the Phylofish online database. (PDF 606 kb)

Additional file 5: Evaluation by qPCR for transcripts of *wnt*, *tnf,* and *nf-kb* pathways in *otulina* mutant-derived eggs. Transcript levels of (a) *tcf3*, (b) *tnfa*, (c) *ikkaa*, (d), *nf-kb2*, (e) *rel*, and (f) *rela* were investigated by qPCR (PDF 76 kb)

## Data Availability

The datasets generated and materials used in the current study are available upon request. In addition, all microarray data are available in NCBI Gene Expression Omnibus under accession GSE109073: https://www.ncbi.nlm.nih.gov/geo/query/acc.cgi?acc=GSE109073.

## Abbreviations

2-FCV: 2-fold cross validation
DEGs: differentially expressed genes
FDR: false discovery rate
GA: genetic algorithm
*gfp*: green fluorescent protein
hpf: hours post-fertilization
MBT: mid-blastula transition
*npm2a* and *npm2b*: nucleoplasmin 2a/b
*otulina*: OTU deubiquitinase with linear linkage specificity a, fam105ba
PLS: partial least square regression
qPCR: quantitative real-time polymerase chain reaction
RNA-seq: RNA sequencing
*rpf2*: ribosome production factor 2 homolog (*S. cerevisiae*)
*rps27.2*: ribosomal protein S27 (isoform 2)
*rtn4ip1*: reticulon 4 interacting protein 1
*slc29a1a*: solute carrier family 29, member 1a
*spon1b*: spondin 1b
*stra13*: stimulated by retinoic acid 13 homolog/centromere protein X
*tspan7b*: tetraspanin 7b
*U1*: U1 spliceosomal RNA
WT: wildtype
ZGA: zygotic genome activation
