# Supplementary figures and images for: What makes a bad egg? Egg transcriptome reveals dysregulation of translational machinery and novel fertility genes important for fertilization
Source: BMC Genomics. 2019 Jul 15;20:584. doi: 10.1186/s12864-019-5930-8 (PMC6631549; doi:10.1186/s12864-019-5930-8)

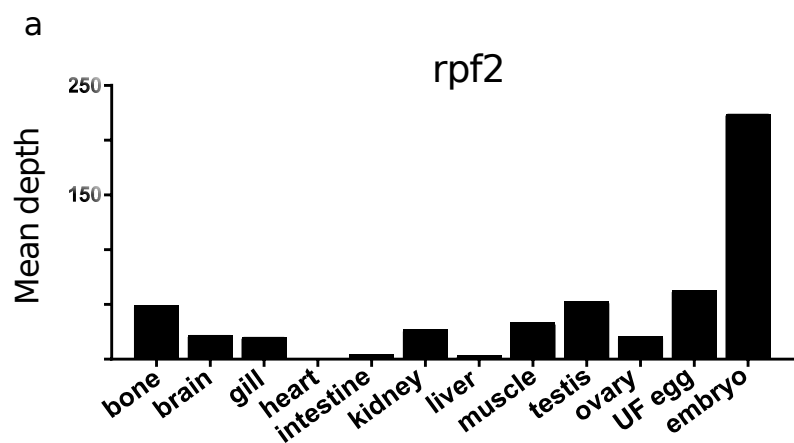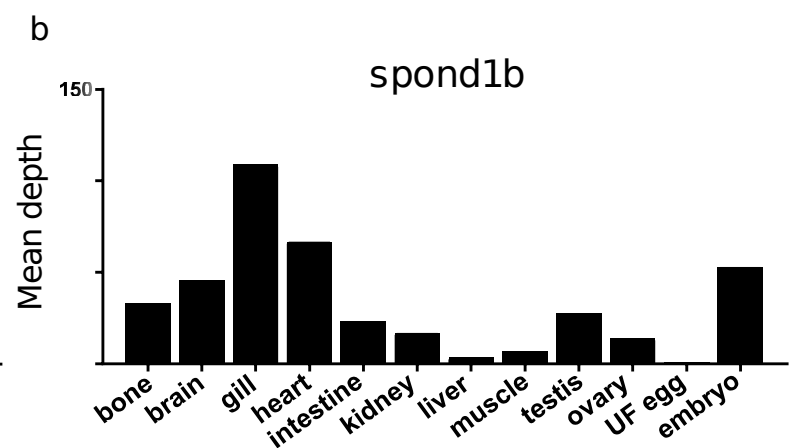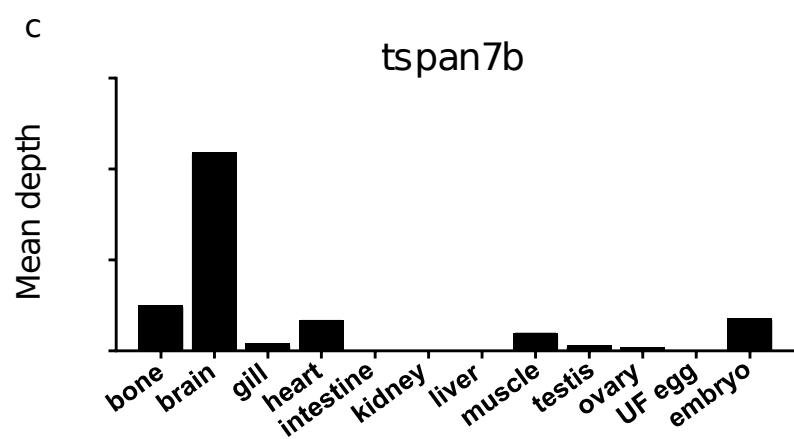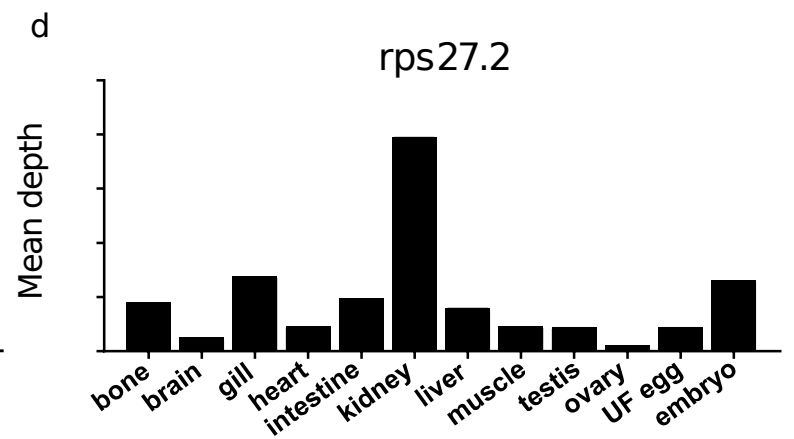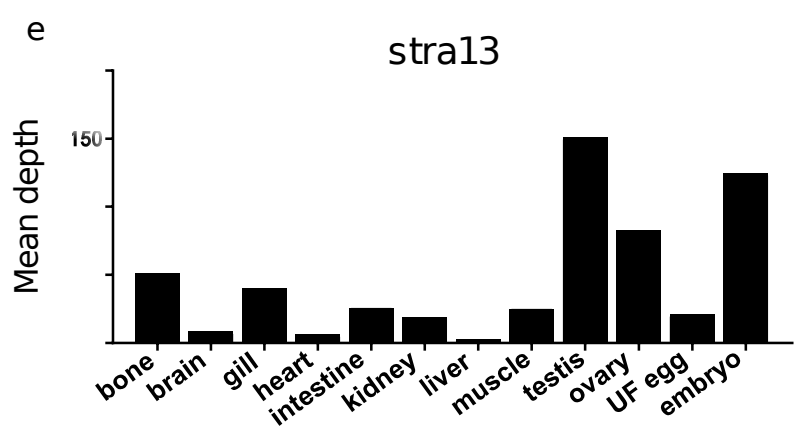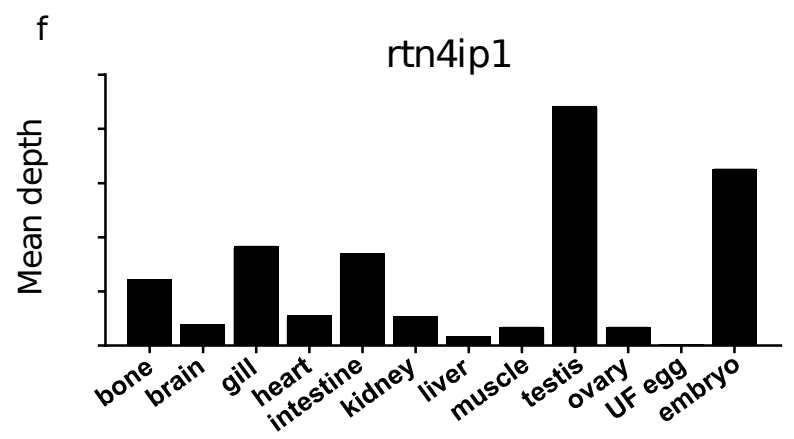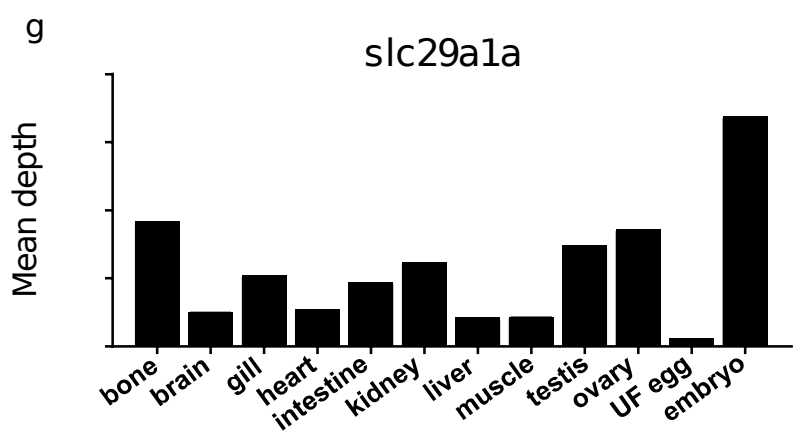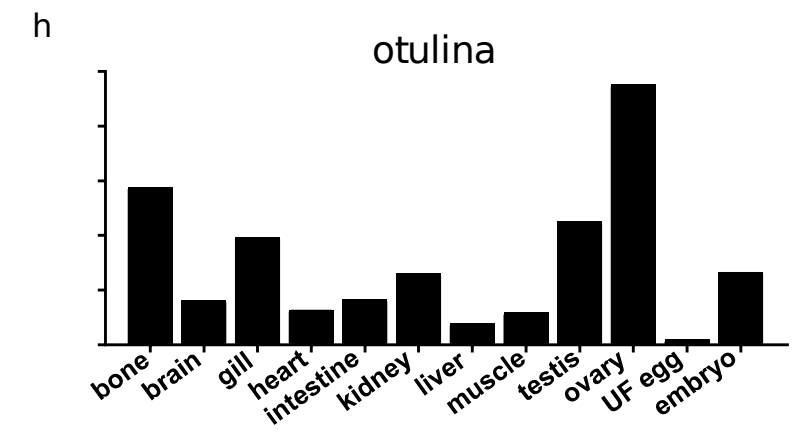

Supplement: Supplementary file 4 — Tissue localization of (a) rpf2, (b) spond1b, (c) tspan7b, (d) rps27.2, (e) stra13, (f) rtn4ip1, (g) slc29a1a, and (h) otulina transcripts by RNA-seq retrieved from the Phylofish online database. (PDF 606 kb) [file 12864_2019_5930_MOESM4_ESM.pdf]

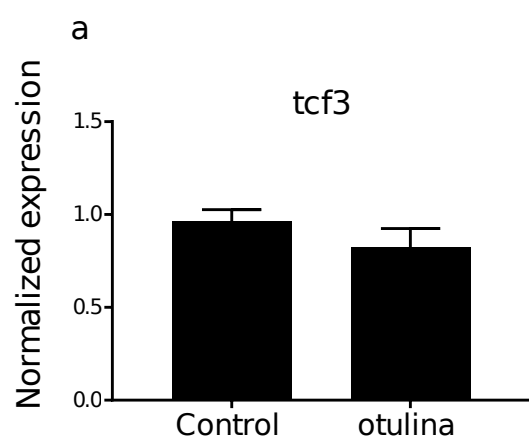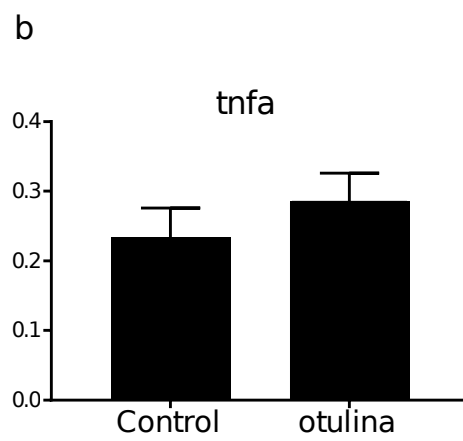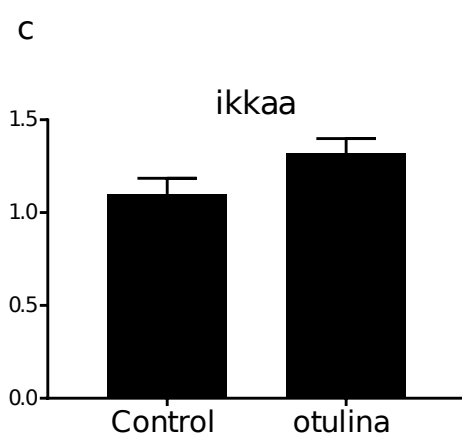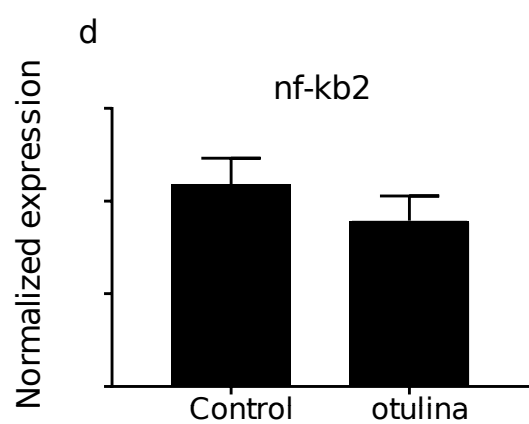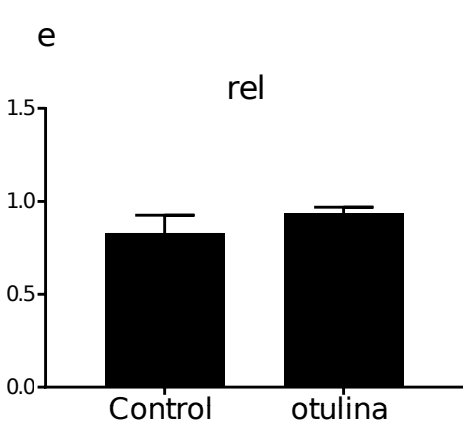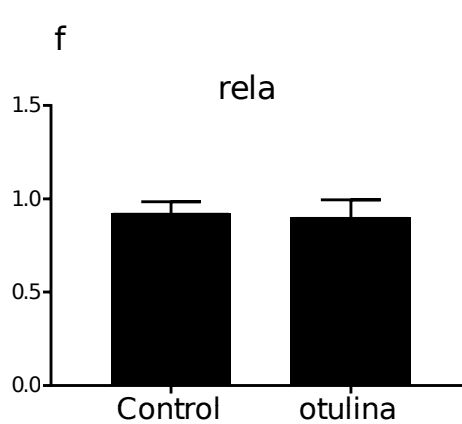

Supplement: Supplementary file 5 — Evaluation by qPCR for transcripts of wnt, tnf, and nf-kb pathways in otulina mutant-derived eggs. Transcript levels of (a) tcf3, (b) tnfa, (c) ikkaa, (d), nf-kb2, (e) rel, and (f) rela were investigated by qPCR (PDF 76 kb) [file 12864_2019_5930_MOESM5_ESM.pdf]
